# Influence of Mn and Co ions co-doping on the photovoltaic performance of CdS quantum dot sensitized solar cells

**DOI:** 10.1038/s41598-025-19834-6

**Published:** 2025-10-14

**Authors:** G. Vinoth, B. Janarthanan, Jhelai Sahadevan, A. Dinesh, Lalitha Gnanasekaran, Manikandan Ayyar, Madhappan Santhamoorthy, S. Santhoshkumar, Prabhu Paramasivam, Sandeep K., Gaurav K.

**Affiliations:** 1 Centre for Sustainable Materials Research, Department of Physics, Academy of Maritime Education and Training (AMET), Deemed to Be University, Kanathur, Chennai, Tamil Nadu 603112 India; 2 Department of Physics, Karpagam Academy of Higher Education, Eachanari Post, Pollachi Main Road, Coimbatore, Tamil Nadu 641 021 India; 3 Centre for Energy and Environment, Karpagam Academy of Higher Education, Eachanari Post, Pollachi Main Road, Coimbatore, Tamil Nadu 641 021 India; 4 Department of Chemistry, K. Ramakrishnan College of Engineering (Autonomous), Affiliated to the Anna University, Samayapuram, Trichy, Tamil Nadu 621112 India; 5 Instituto de Alta Investigación, Universidad de Tarapacá, 1000000 Arica, Chile; 6 Department of Chemistry, Karpagam Academy of Higher Education, Coimbatore, Tamil Nadu 641 021 India; 7 Centre for Material Chemistry, Karpagam Academy of Higher Education, Tamil Nadu, Coimbatore, 641 021 India; 8 School of Chemical Engineering, Yeungnam University, Gyeongsan, 38541 Republic of Korea; 9 Department of Biochemistry, Saveetha Medical College and Hospital, Saveetha Institute of Medical and Technical Sciences, Chennai, Tamil Nadu India; 10 Centre for Research Impact & Outcome, Chitkara University Institute of Engineering and Technology, Chitkara University, Rajpura, Punjab 140401 India; 11 Department of Mechanical Engineering, Ramaiyah University of Applied Science, Bangalore, Karnataka India; 12 Research and Innovation Cell, Delhi Technological University, Delhi, New Delhi India; 13 Department of Mechanical Engineering, Mattu University, 318 Mettu, Ethiopia

**Keywords:** Mn-doped CdS QDs, Dual doping (Mn/Co), Quantum confinement effect, QDSSCs, Power conversion efficiency (PCE), Materials science, Physics

## Abstract

The co-precipitation method was used for the synthesis of CdS quantum dots doped with Mn (1%, 2%, and 3%) and Mn (1%)/Co(2%) and Mn(2%)/Co(4%). Powder X-ray diffraction (XRD), transmission electron microscopy (TEM), UV–Vis absorption spectroscopy and photoluminescence (PL) spectroscopy analysis was carried out and evaluated their structural, morphological and optical properties. The quantum dot sensitized solar cell with the incorporation of the samples in photoanode is subjected for J-V characteristics to determine the solar cell parameters. Cubic structure of Mn and Mn/Co co-doped CdS quantum dots was obtained with the grain size of 10 nm confirmed by TEM images. The energy bandgap (E_g_) values are varying between 2.98 and 2.89 eV for Mn/CdS and Mn/Co co-doped CdS, which was confirmed from Tauc plot. The maximum power conversion efficiency (1.67%) was obtained for the solar cells Mn (1%) and Co (2%) co-doped CdS with fill factor (*ff*), open circuit voltage and short circuit current density of 0.67, 0.3703 V and 6.7365 mA/cm^2^, respectively.

## Introduction

Cadmium sulfide (CdS) is an n-type semiconductor exhibits two crystal forms namely hexagonal (wurtzite) and cubic (zinc-blende). It has a direct band gap of 2.4 eV and making it useful in optoelectronic devices, including lasers, LEDs, and solar cell photodetectors. The optical properties of CdS quantum dots, including absorbance, transmittance, reflectance, and luminescence properties are depends of the crystal structure and grain size. CdS quantum dots can be synthesized by various methods including the spray pyrolysis, pulsed laser deposition, co-precipitation, thermal evaporation, sputtering, and chemical vapor deposition, etc. CdS quantum dots doped with transition metals like Mn, Co, Fe, and Ag etc. have been changed their electrical, optical, and magnetic properties, because of the incorporation of the CdS electron band and the localized transition metal electrons in the d-orbitals.

Doping of Co-ions in the CdS quantum dots leading to the generation of a high density of point defects including, antisite defects, interstitials, and vacancies in the CdS forbidden band gap [1]. It is necessary to determine the influence of dopant concentration on the optical and electrical properties of the materials. It has been discovered that the chemical precipitation technique has several benefits, including ease of processing under ambient conditions, the ability to dope various impurities with high concentrations of dopant even at room temperature, ease of surface capping, and good control over the chemistry of doping with some different metal ions for the formation of nano-particles [2]. Doped materials display a variety of unique forms of luminous features, the precise nature, which is strongly dependent on the types of dopant ions in the material. These dopant impurities are responsible for a major portion of the electrical and structural change that is brought about in the host materials, as well as the transition probabilities between states [3].

Introducing impurity or dopant ions/atoms into the lattice structure of a pure semiconductor led to the tuning their physical, chemical and electrical characteristics to a larger extent. This process, which creates new states in the band gap area of the semiconductor, is more often referred to as doping [4]. Metal ion doping is commonly utilized to increase efficiency of the materials. Better morphological development of the materials will be the greatest support for increasing photocatalytic activity [5]. Recent studies have focused on enhancing performance and reducing material costs in QDSSCs and other photonics technologies [6]. Numerous research projects on redox couples focus to suppress the recombination reaction that takes place at the nanostructured TiO_2_/redox electrolyte interface between the injected electrons and the oxidized molecule of the redox electrolyte to increase the open circuit photovoltage and short-circuit current density [7]. The construction of QDSSCs, instead of organic dyes, wide bandgap semiconductor quantum dots like PbS, CdSe, or CdS are formed over mesoscopic wide bandgap oxide thin films, usually TiO_2_ or ZnO [8], which are good results than others.

Though the QDSSCs has shown lesser efficiency, it overcomes the degradability and sustainability compared to the third-generation DSSCs. The dye degradation in DSSCs led to further experimentation to increase the sustained performance. In this context, the QDSSCs has its significance in sustainable performance and environmental stability. Further in QDSSCs, it is possible to tune the bandgap for the effective photo response for multiple exciton generation by considering the dopants. Also, the quantum dots have higher molar extinction coefficients, which absorbs more light per unit concentration falling on it. In the present work, an effort has been taken to synthesize the CdS quantum dots with Mn and Co-codopant to tune the bandgap of CdS to absorb more light for the generation of excitons. The quantum dots are subjected for optical, structural, morphological and electrical studies through UV–Visible spectra, photoluminescence spectra, XRD, TEM and J-V characteristics study.

## Materials

Chemicals of analytical grades such as cadmium nitrate, sodium sulfide, manganese nitrate, cobalt nitrate, PVP, and ammonia solution were utilized without further purification, and deionized water served as a solvent throughout the whole procedure. For the construction of solar cell devices, FTO substrates were used.

### Synthesis of CdS quantum dots and Mn/Co co-doped CdS quantum dots

Followed with the synthesis and characterization of pure CdS [9], the co-precipitation technique was used for the preparation of Mn-doped CdS and Mn/Co dual-doped CdS QDs. Cd(NO_3_)_2_ and Na_2_S were obtained in separate quantities of 0.1 mol and 0.9 mol and suspended in 50 mL of deionized water. In order to get a homogeneous mixture, PVP was added to Cd(NO_3_)_2_ and stirred for 15 min followed by the agitation of the mixture for 30 min. Na_2_S was added dropwise to the Cd(NO_3_)_2_ + PVP mixture to get the required precipitate. The obtained precipitate was cleaned and dried for 12 h at 80 °C. By substituting 0.1 mol of cadmium nitrate with manganese acetate and cobalt nitrate, two distinct solutions for Mn:CdS and (Mn/Co):CdS quantum dots were obtained. In order to doping of Mn with CdS:Mn QDs, 1%, 2%, and 3% of manganese acetate were added to Cd(NO_3_)_2_ at the beginning of the process. The precipitate was grinded to a fine powder using a mortar and pestle once it dried completely. The same process has also been used to dope (Mn + Co) into cadmium nitrate in order to obtain CdS:Mn + Co. The ratios of dopant concentrations and sample codes were mentioned in the Table 1.

**Table 1:** Synthesis of single (Mn) and dual (Mn/Co) doped CdS QDs, with the concentrations and doping percentages.

Sample code	Precursors	Dopant concentrations	Solvent
A	CdS: Mn	Mn (1%)	DI
B	CdS: Mn	Mn (2%)	DI
C	CdS: Mn	Mn (3%)	DI
D	CdS: Mn/Co	Mn (1%) / Co (2%)	DI
E	CdS: Mn/Co	Mn (2%) / Co (4%)	DI

### Device fabrication

10 drops of nitric acid followed by the addition of 10 drops of acetic acid and added 0.5 g of TiO_2_ to obtained a paste of TiO_2_. The TiO_2_ is coated over FTO substrate having the sheet resistivity of 7 Ω/square by Doctor Blade method. Followed, Mn/CdS and Mn/Co co-doped CdS quantum dots was made into paste using ethanol. The paste of the Mn and Co co-doped CdS is coated over TiO_2_ coated FTO substrate and served as the Photoanode. The counter electrode of graphene coated FTO is sandwiched to the photoanode using binder clip. The electrolyte solution of (I^-^/I^-^3) is prepared using milli-Q water, potassium iodide, iodine, and acetonitrile were dissolved and stirred for 30 min. A few drops of the electrolyte solution were added in between the interfacial layers of photoanode and counter electrode to form the complete solar cell. The architecture of the fabricated solar cells FTO/TiO_2_/Mn doped CdS/I^-^/I^-^_3_/graphene/FTO and FTO/TiO_2_/Mn/Co co-doped CdS/I^-^/I^-^_3_/graphene/FTO was used for further analysis.

## Results and discussion

### XRD analysis

**Fig. 1:**
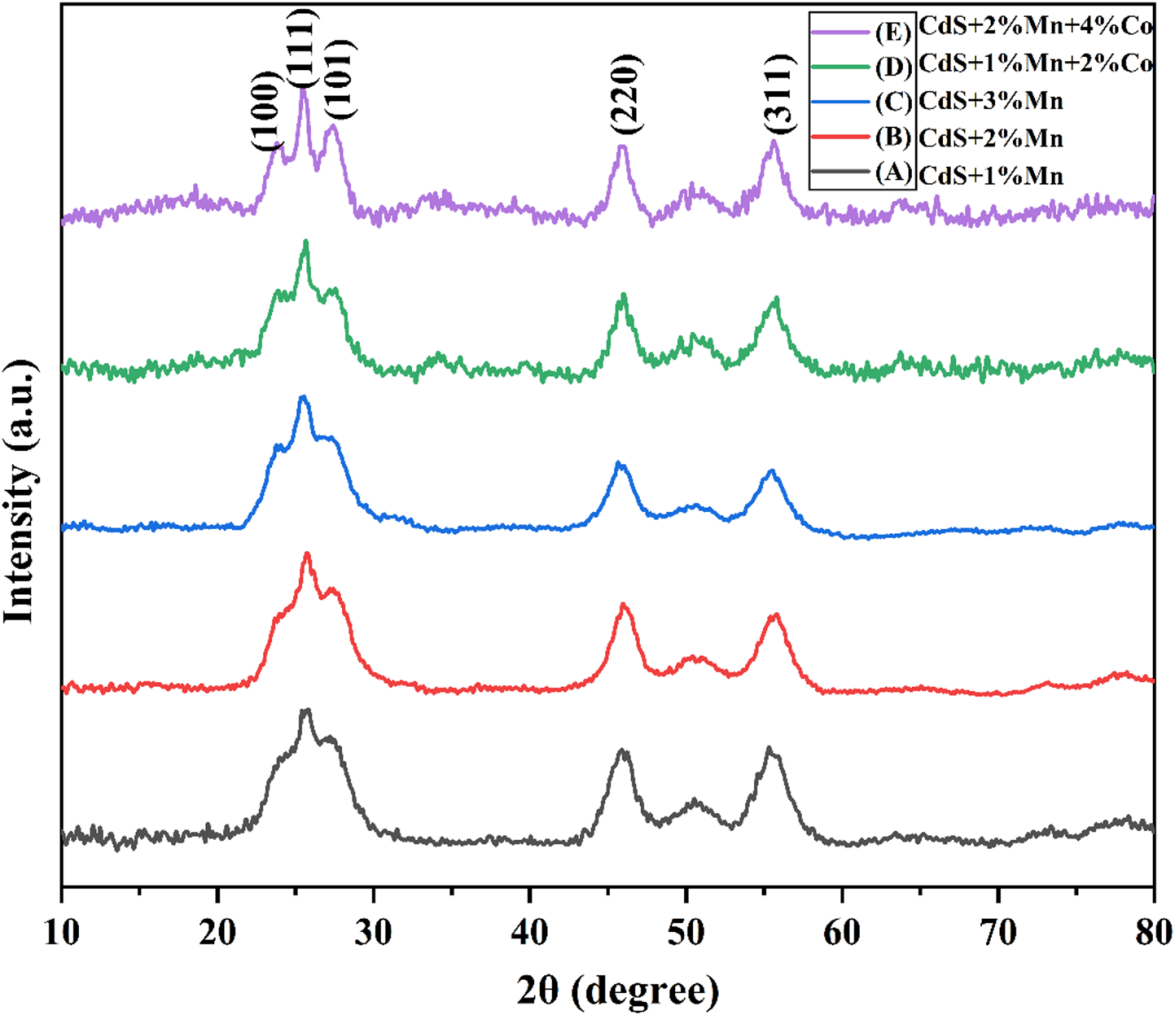
XRD patterns of Mn-doped CdS QDs and Mn/Co doped CdS QDs with increasing dopant concentrations.

XRD analysis was used to determine the size, crystallinity, and phase of the synthesized samples as shown in Fig. 1. Doped CdS quantum dots with Mn and Co display three XRD peaks that correspond to the (111), (220), and (311) planes, but CdS quantum dots show two additional peaks that correspond to the (100) and (101) planes respectively. The phase purity of the films is shown by the observed diffraction peaks, which are in good accordance with JCPDS standard card 89–0440 and cubic phase without any additional impurity phases. The significant widening of the (111) plane indicates the nano-phase development occurred. Mn doping shifts the peak to a higher angle, implying lattice compression. It may be inferred that there is no change in the crystal structure of CdS after doping Mn, since there is no noticeable change in the XRD pattern of CdS. Another potential explanation for the decrease in XRD peak intensity is the lower atomic scattering factor of Mn^2+^ compared to Cd^2+^. Sun et al*.* found the similar pattern in Mn-doped CdS [10]. During the process of doping cobalt into CdS, the preferred orientation plane (111) moves towards the higher angle side. This indicates that cobalt ions replace cadmium sites without affecting the crystalline structure of the CdS. Aksay et al*.* observed the same shift towards the 2θ side caused by Co-doping into CdS [11].

The following Eq. (1) was used to estimate the crystallite size of the samples,

1 $$D=\frac{K\lambda }{\beta cos\theta }$$

where, D is the average crystallite size, K is a constant, λ is the wavelength of X-Ray (1.5406 Å), β is the full width at half maximum (FWHM) in radians, and θ is the Bragg’s diffraction angle, respectively. The calculated vales are tabulated in the Table 2, from the results it was observed that the crystallite sizes are increased, because the ionic radius of Co^2+^ (0.74 Å) [12] and Mn^2+^ (0.66 Å) [13] is less than that of Cd^2+^ (0.96 Å), it is confirmed that both Co^2+^ and Mn^2+^ are cadmium replacements by the fact that the crystallites size was increased by Co and Mn doping. Further the peak obtained at 51°, confirms the presence of dopants in CdS lattice, due to the formation of secondary phases of the dopant atoms led to the stress within the lattice.

**Table 2:** Structural parameters of Mn and Mn/Co doped CdS QDs.

Sample code	2θ position	FWHM (β)	Crystallite size (nm)	Micro-stain (ε) × 10^–3^	Dislocation density (γ) × 10^–3^ (nm^-2^)
A	25.7	1.197	7.1	19.6	21.09
B	25.9	1.132	7.5	17.5	19.82
C	25.4	0.906	9.3	11.3	17.46
D	25.5	0.887	9.5	10.8	17.15
E	25.5	0.865	9.8	10.3	16.62

It is clear that, Mn and Co co-doping on CdS affect the crystal structure leading to the mixed phase of cubic and hexagonal structure. The defects in the crystal lattice of CdS, due to doping will be acting as centers for trapping and recombination of charge carriers. The change in size and shape of the particles size is due to the stimulus effect of the Mn and Co for the growth and morphology of CdS quantum dots. Further, the doping of Mn and Co led to the additional energy levels in the bandgap of CdS to smoothen the electron or hole transfer in correlation with the conduction or valence band of CdS quantum dots. The introduction of energy levels in the bandgap of CdS results in the enhanced separation of photo-generated charge carriers more effectively for the charge transport towards the opposite electrode in the solar cell device.

### Morphology analysis

**Fig. 2:**
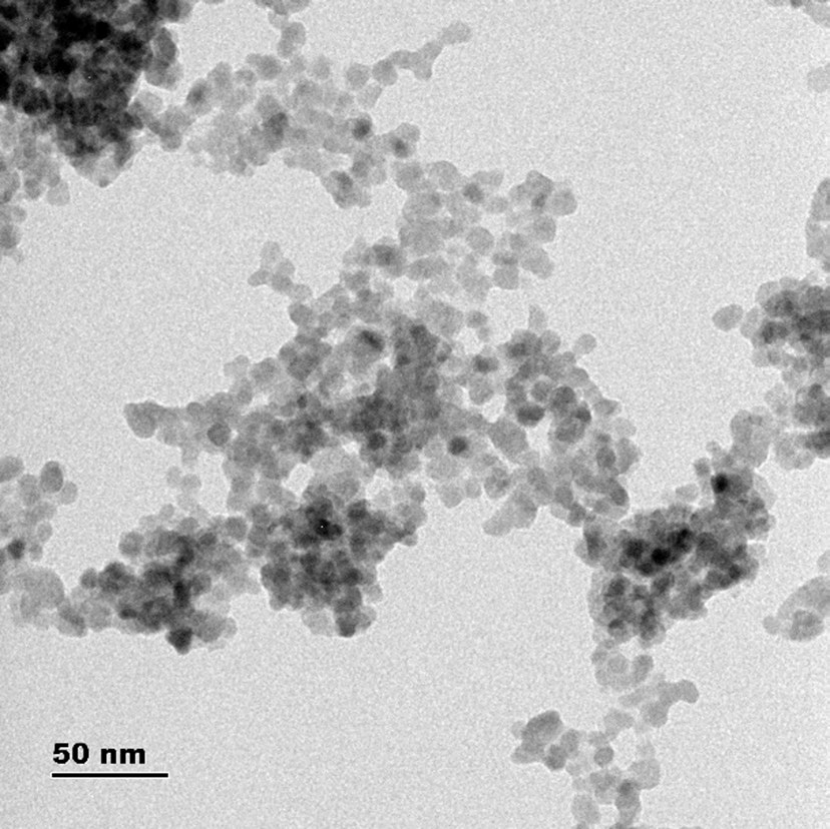
HR-TEM photograph of bare CdS quantum dots.

The synthesis, characterization of pure CdS and their comprehensive analysis was reported earlier by the authors [9]. HRTEM analysis of the pure CdS are shown in the Fig. 2. It is clear that the size of the quantum dot varies from 7.5 nm to 9 nm. Researches have inferred the Bohr exciton radius of CdS quantum dots is 5.8 nm, whereas the size of CdS quantum dots with the dopant is greater than the bare CdS. Also, there is no significant change in the value of the bandgap of CdS quantum dots with the dopants.

**Fig. 3:**
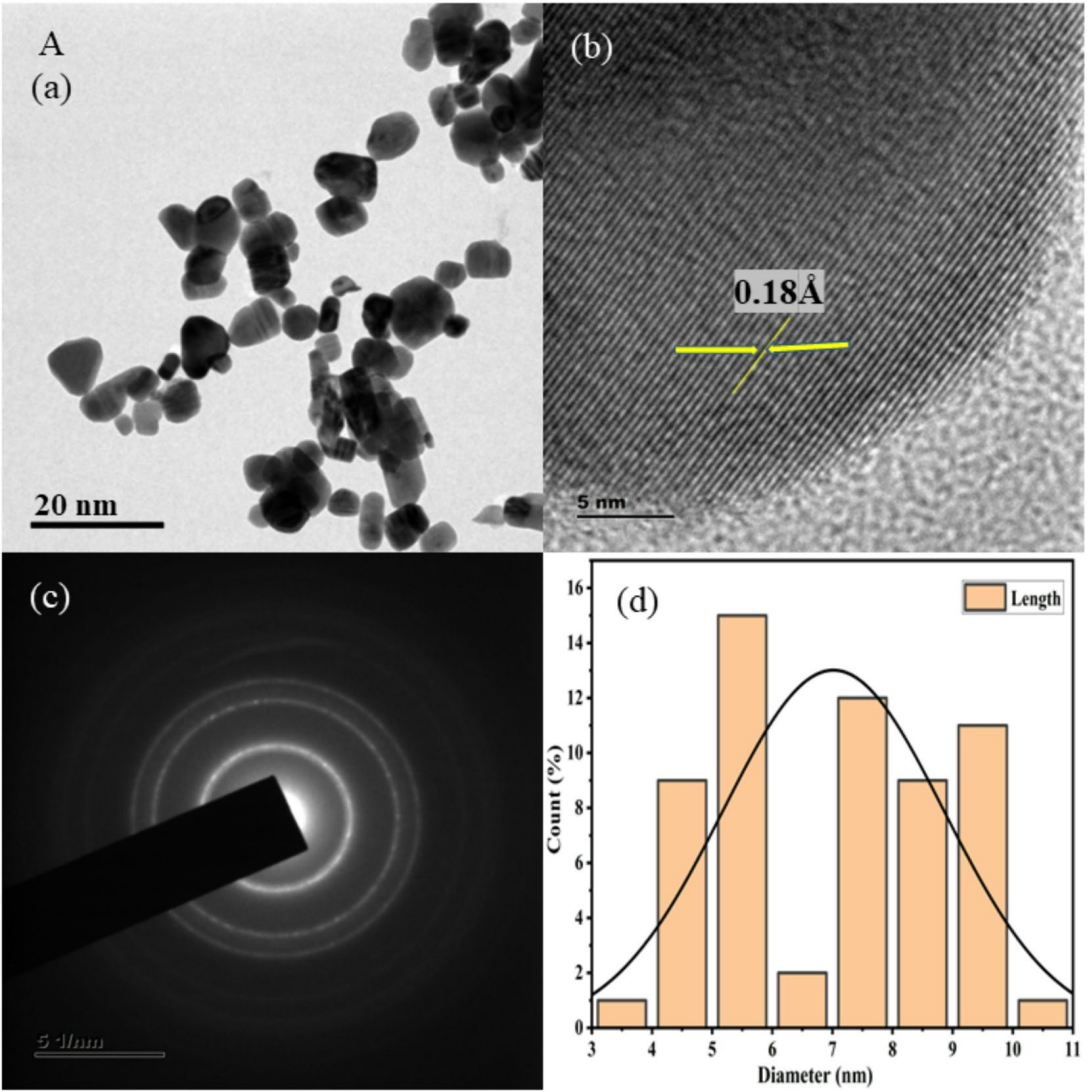
HRTEM images (**a**, **b**), SAED patterns (**c**), and histogram plots (**d**) of Mn (1%) doped CdS QDs.

**Fig. 4:**
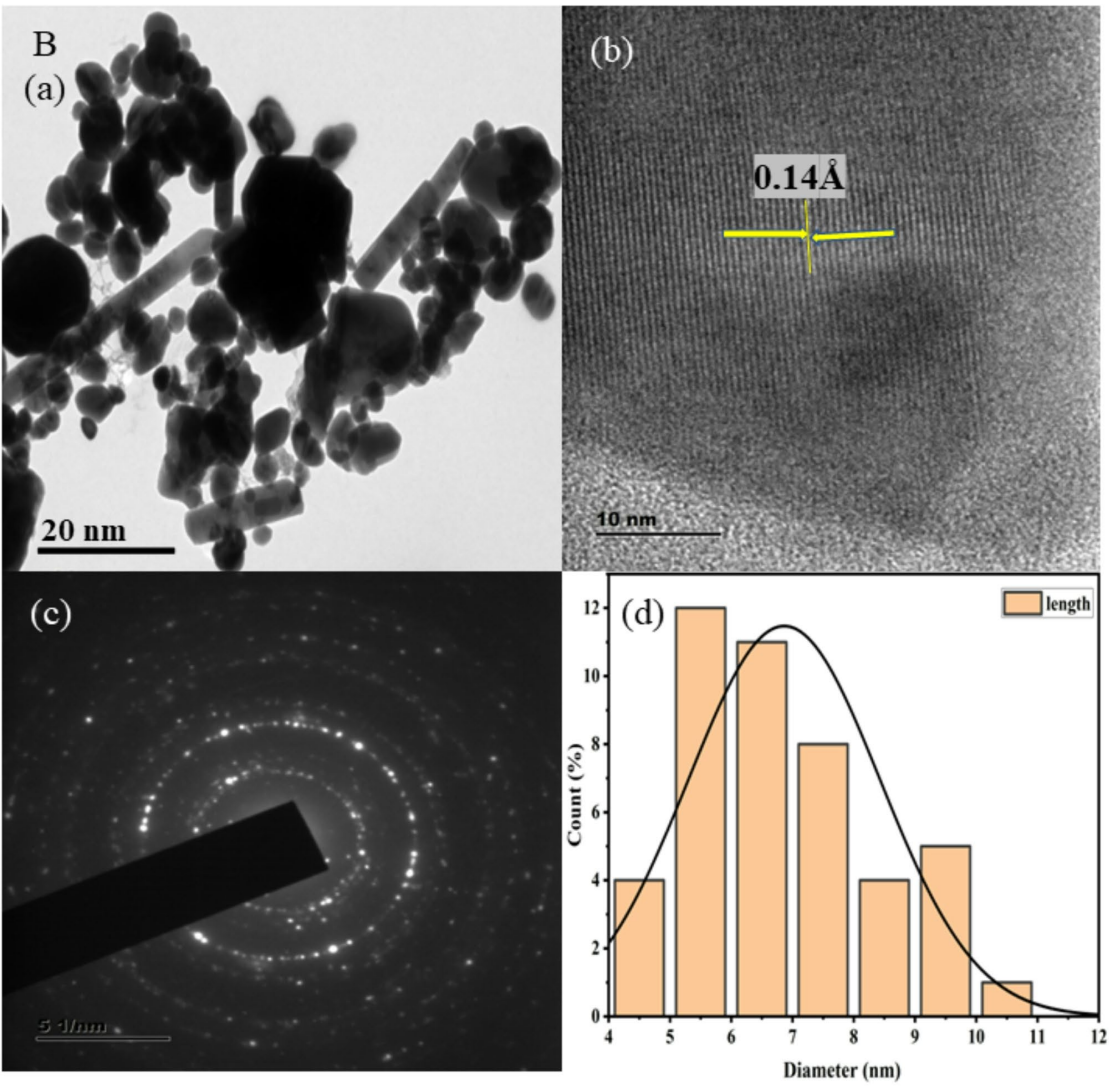
HRTEM images (**a**, **b**), SAED patterns (**c**), and histogram plots (**d**) of Mn (2%) doped CdS QDs.

**Fig. 5:**
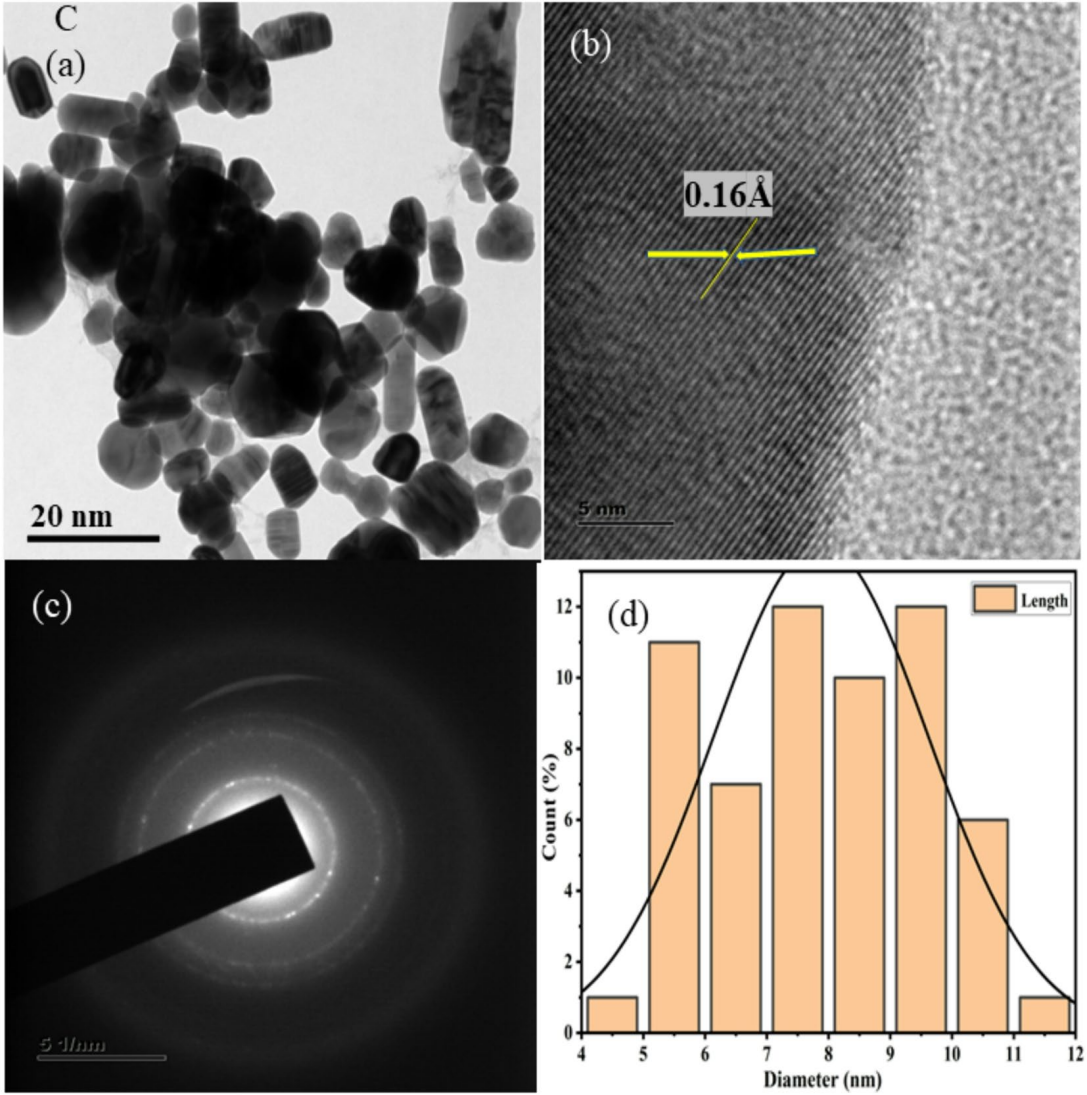
HRTEM images (**a**, **b**), SAED patterns (**c**), and histogram plots (**d**) of Mn (3%) doped CdS QDs.

**Fig. 6:**
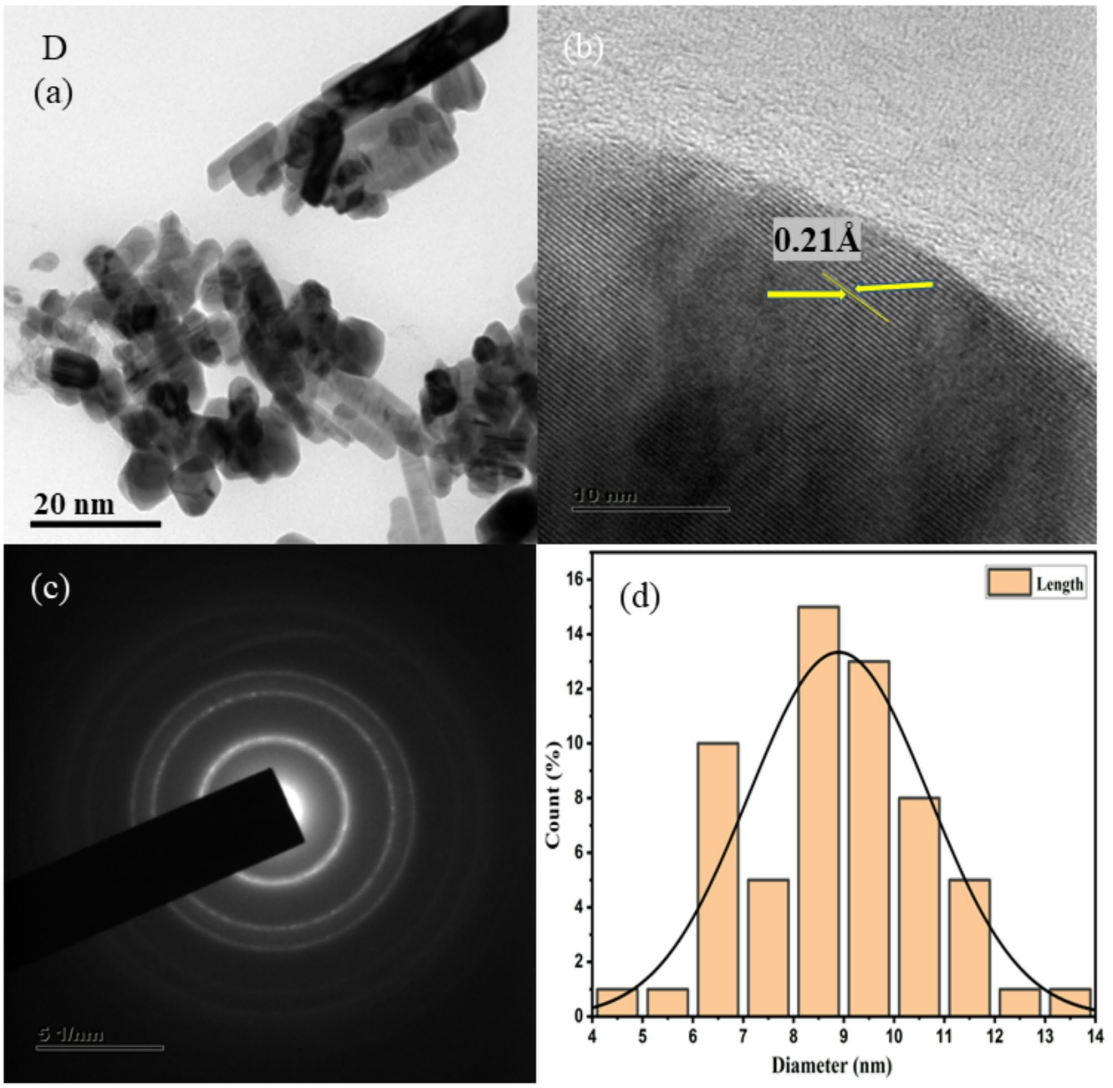
HRTEM images (**a**, **b**), SAED patterns (**c**), and histogram plots (**d**) of Mn (1%)/Co (2%) co-doped CdS QDs.

**Fig. 7:**
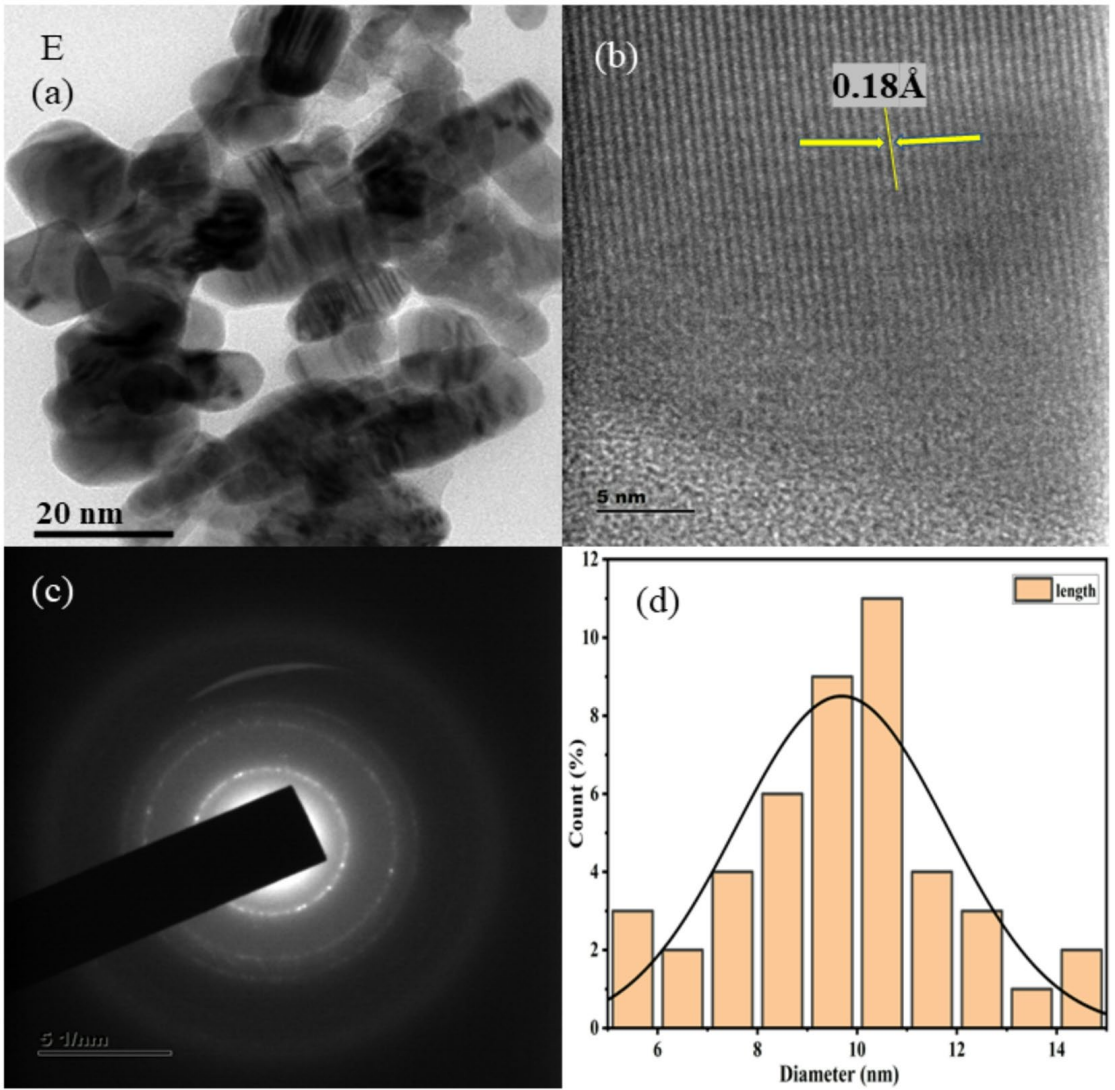
HRTEM images (**a**, **b**), SAED patterns (**c**), and histogram plots (**d**) of Mn (2%)/Co (4%) co-doped CdS QDs.

HR-TEM images at low and high magnification are shown in Figs. 3, 4, 5, 6, 7, together with corresponding selected area diffraction (SAED) patterns, d-spacing values, and histogram analysis of Mn and Mn/Co co-doped cadmium sulfide quantum dots. The presence of bright spots in the rings and the SAED pattern both assure that the particles are of high crystalline order. The particle sizes of doped CdS QDs are increasing consistently, and they are in good agreement with the XRD data. Higher-magnified images of co-doped CdS quantum dots exhibit lattice fringes associated with (111) planes, indicating good crystalline character. It was easy to add two different cations (Mn^2+^ and Co^2+^) to the CdS lattice, and the particles are on the same scale as quantum dots in the range from 7 to 10 nm as shown in Fig. 3. In addition, the d spacing values are measured as 0.18 Å, 0.14 Å, 0.16 Å, 0.21 Å, and 0.18 Å, and the lattice fringes are consistent and free of disorientations and dislocations. This leads one to conclude that the inclusion of Mn and Co did not result in any lattice strain. The TEM analysis was done using the ImageJ software and the diameter of all the particles are found to draw the histogram to determine the average particle size. The d-spacing for the corresponding TEM image is evaluated using ImageJ software.

### UV–Vis analysis

**Fig. 8:**
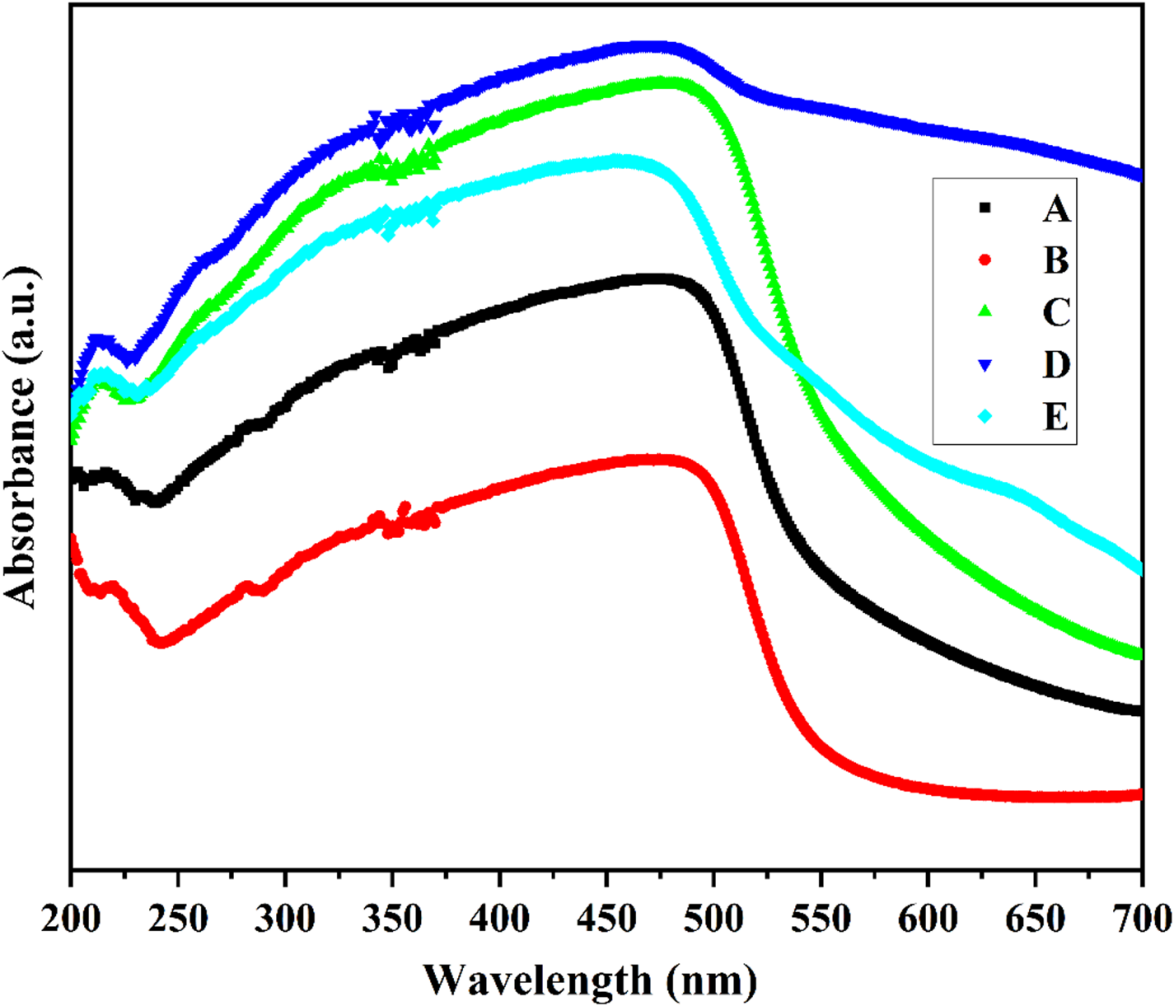
UV–vis absorbance spectra of prepared Mn: CdS QDs and (Mn/Co): CdS QDs at different dopant concentrations are represented as A, B, C, D, and E.

**Fig. 9:**
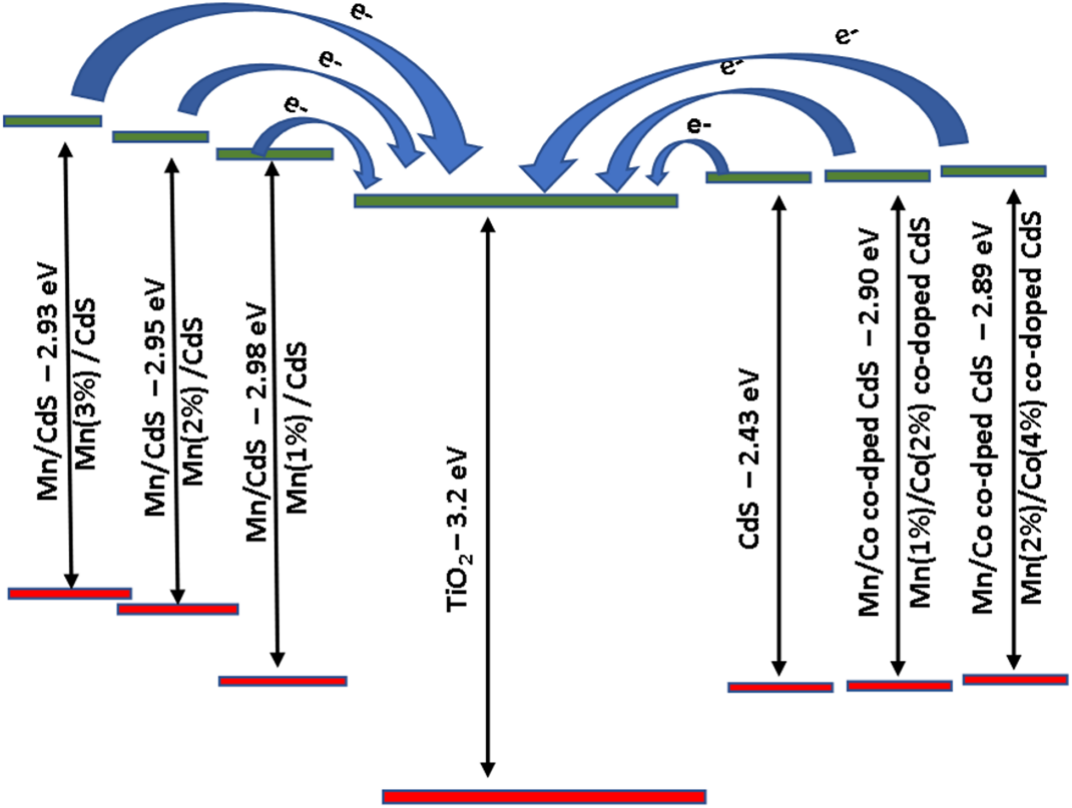
Energy gap diagram for pure, doped and co-doped CdS quantum dots.

UV–Vis absorption spectra of Mn and Mn/Co co-doped CdS QDs are shown in Fig. 8 followed with the energy gap diagram also depicted in Fig. 9. The absorption edges of Mn and Co-doped CdS samples are 496,492,490,485 and 483 nm, which are slightly blue-shifted from the absorption edge of CdS quantum dots at 510 nm [2]. Optical absorption studies have suggested that the absorption edge moves toward the blue region compared to bulk samples of CdS. This means that the effective band gap energy decreases as the size of the particle increases. The blue shift in the excitation wavelength is thought to be generated by the quantum confinement effect in bulk materials with these compositions [14]. The absorption edges are about the same for all samples and exhibit blue shift due to doping. As electrons are transferred from the valence band (VB) to Fermi energy levels in the conduction band (CB), the absorption edge increases. This outcome might be attributable to the effective low proportion of dopants [3]. The quantum size effect is responsible for the blue shift in the excitonic absorption peak, but the widening and asymmetry are caused by the wide size dispersion of synthesized particles. When excitonic peaks from various sizes of particles overlap at different energies, the absorption spectra are widened [15, 16]. The band gap of the material is established using the fundamental absorption, which corresponds to transmission from the valence band to the conduction band.

From Fig. 9, it is clear that, the absorption bandgap of CdS quantum dots with Mn and Co co-doped CdS samples are shows a minimum energy gap of 2.89 eV (Mn (2%) and Co (4%)). It is inferred that the doping concentration influences the energy gap and new energy level is introduced near to the conduction band edge of TiO_2_ leading to effective charge transfer.

Tauc plots between (αhv)^2^ and energy are drawn, and the energy band gap was calculated by extrapolating the linear component of the curve to the energy axis. Quantum dots (QDs) have an optical bandgap energy that may be determined using the relation (2),

2 $${\alpha h\nu }^{n}=A(h\nu -{E}_{g})$$

where E_g_ is the material’s band gap and A is a constant. The values of the exponent "n" are 1/2, 2, 3/2, and 3 correspond to the allowed direct, allowed indirect, forbidden direct, and forbidden indirect transitions, respectively [9, 17].

**Fig. 10:**
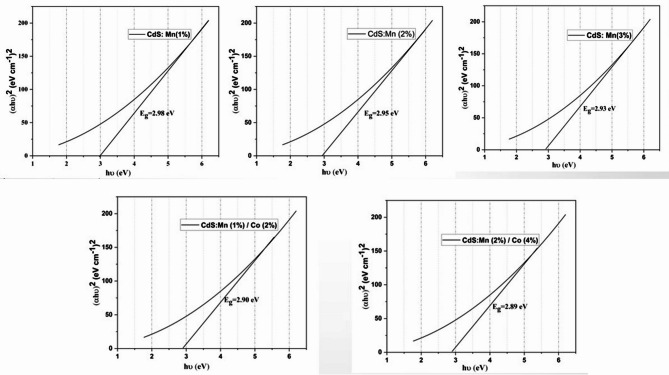
Tauc plot for the Mn and Co co-doped CdS quantum dots.

Figure 10 represents the Tauc plot for all the samples utilizing the UV–Visible spectrum and evaluated the bandgap energy respectively. According to the optical findings, Co and Mn-doped CdS film shows a desired lowered band gap energy and thus can absorb more sunlight across a wide spectrum area to improve light harvesting capabilities [18] and the band gap values of samples A, B, C, D, and E are found to be 2.98 eV, 2.95 eV, 2.93 eV, 2.90 eV, and 2.89 eV respectively. Due to variations in electro-negativity and ionic radii, additional defects are likely generated when Mn and Co atoms concurrently replace Cd and S atoms, in their lattice, which results in a decrease in the band gap values [19]. The low energy border of the basic absorption, also known as the absorption edge, is often the most noticeable aspect of the spectrum in semiconductors [4].

### PL analysis

**Fig. 11:**
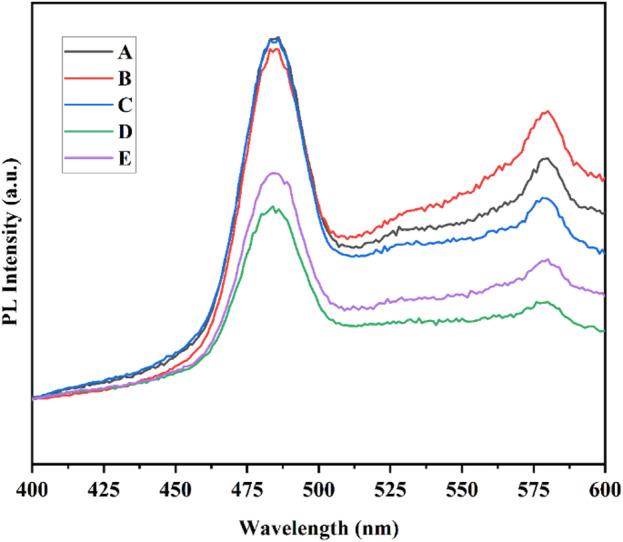
Photoluminescence spectra of single and dual-doped CdS QDs with increasing dopant concentrations are represented as A, B, C, D, and E.

Photoluminescence (PL) spectra of CdS: Mn and CdS: Mn/Co co-doped CdS samples are shown in Fig. 11. The PL spectra shows two prominent peaks in which the excitation was done starting from 400 nm as wide absorption peak is obtained at 400 nm in UV–Visible spectrum. PL spectra for various dopant concentrations of Mn and Co are shown in Fig. 11. The peak is obtained at 484 nm and the other peak is found at 579 nm. A monochromatic photon beam of certain energy excites an electron, which then undergoes either at the valence band or by radiative recombination (band edge luminescence) or at traps/surface states (often red-shifted luminescence) inside the forbidden gap [4]. In CdS, the possible defects are Cd and S vacancies, and Cd and S interstitials [20]. The examination of the PL spectrum enables the identification of particular defects or impurities, and the strength of the PL signals enables the quantification of their concentration. Mn doping does not cause a change in the emission band; nevertheless, the intensity of the band does rise with increasing Mn concentration. On the other hand, when there is a rise in the concentration of Mn, the emission band moves toward a shorter wavelength [4]. The reduced rate of electron–hole pair recombination may be the reason for the decrease of PL intensity [21].

The increment in the concentration of Mn and Co in the CdS sample enhanced the PL intensity of Cd_1-x_Mn_x_S quantum dots synthesized by the chemical co-precipitation technique [22]. The synthesis process and the type of doping material have an impact on the PL intensity. The significant and wide green band emission was produced by the recombination of surface states, which involves electrons trapped in a sulfur vacancy and a hole in the cadmium sulfide valance band. Cobalt/manganese doping enhanced the population of surface Cd^2+^ sites (as interstitials), which perform as electron trap sites, and sulfur vacancy S^2-^ sites, which decrease with the reduction of crystallite size [23]. The colors blue, green, and yellow-green were produced by a cadmium sulfide quantum dot that was prepared by using the co-precipitation method [24]. The benefit or goal of increasing PL intensity is to understand the significant advantages for Mn-Co doped cadmium sulfide quantum dots and to transfer more energy (photons) by a wavelength. This is accomplished by increasing the recombination of an electron constrained inside a sulfur vacancy with a hole in the valance band, which results in increased PL intensity [25].

### Photocurrent to photovoltage studies (J-V)

Keithley 2450 electrometer with the simulator with AM 1.5G solar simulator with the input power of 100 mW/cm^2^ has been used to measure J-V characteristics of the fabricated devices in order to evaluate the solar cell properties. The short circuit current density (J_sc_), maximum current density (J_m_), open circuit voltage (V_oc_), and maximum voltage (V_m_) are obtained from the J-V curves. Using the following Eq. (3) and (4), fill factor and power conversion efficiency (PCE) have been calculated;

3 $$FF=\frac{Jm\times Vm}{Jsc\times Voc}$$

4 $$\eta \%=\frac{FF\times Jsc\times Voc}{Input power}\times 100\%$$

**Fig. 12:**
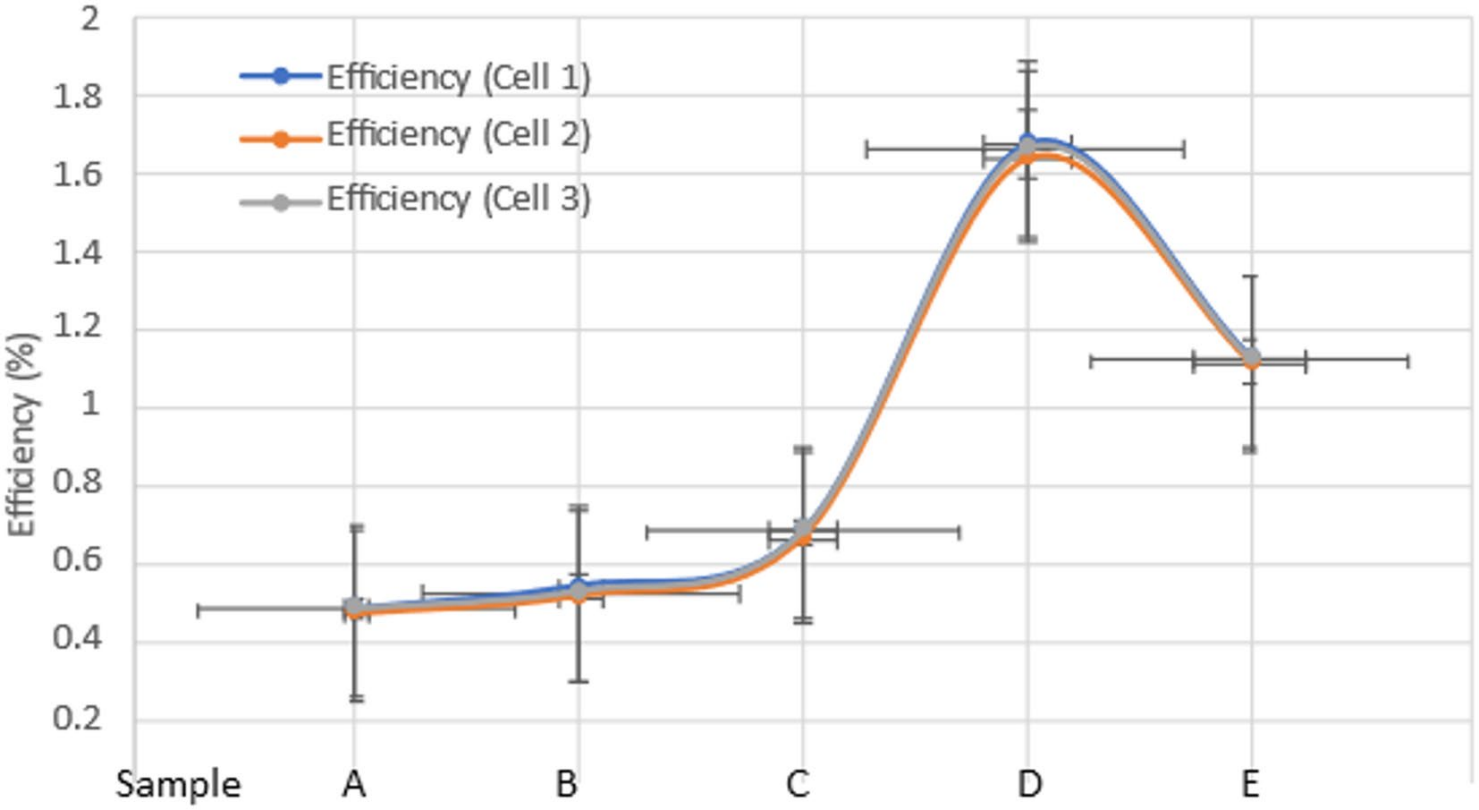
Error bar diagram for the efficiency of three solar cells each with sample A, B, C, D, E.

In Table 3, the photovoltaic properties of various devices are given. All the devices exhibit a clear photovoltaic nature, and the sample D has the highest efficiency, with J_sc_ values of 6.7364 mA/cm^2^, V_oc_ values of 0.3703 V, and FF values of 0.6705, corresponding to a PCE of 1.675%. With increasing Mn and Co doping concentrations, the short circuit current density (J_sc_) and efficiency simultaneously decrease. As shown in Table 4, the PCE of Mn and Co dual-doped CdS QDSSCs improved by nearly 72% (from 0.4801 to 1.675%) when compared to the respective other samples. It is mainly due to the doping concentration was increased, manganese and cobalt ions produce an overabundance of mid-gap states [26]. This results in the trapping of electrons, which blocks the passage of electrons from the quantum dots to the oxide. As a consequence, fewer electrons are able to reach the oxide and their trapped mid-gap energy levels result in a lower current density. Figure 12 represents the error bars of the solar cells fabricated and tested for three solar cells each with samples A, B, C, D and E respectively. The standard deviation between the efficiency of the solar cells has been found and it varies from 0.0050 to 0.106. The current density and efficiency of Mn and Co co-doped CdS quantum dot solar cells decrease, due to electron entrapment, which affects redox operation [8, 27]. When compared to earlier studies based on CdS QDSSCs and to a comprehensive comparison of all the photovoltaic characteristics provided in Table 5, the achieved efficiency of 1.6750% is much higher than those of the earlier works.

**Table 3:** Photovoltaic parameters of single and dual-doped CdS QDs with different dopant concentrations.

Sample code	Open-circuit voltage (V_OC_) [V]	Short-circuit current density (J_SC_) [mA/cm^2^]	Fill factor (FF)	Power conversion efficiency (η%)
A	0.4469	2.9079	0.4749	0.6171
B	0.5595	3.2860	0.5470	1.0056
C	0.4830	3.9574	0.3560	0.6810
D	0.3703	6.7365	0.6705	1.6750
E	0.4410	1.1194	0.7350	1.1223

**Table 4:** Standard deviation for the efficiency of the solar cells.

Sample code	Efficiency (Cell 1)	Efficiency (Cell 2)	Efficiency (Cell 3)	Standard deviation
A	0.4801	0.4657	0.4785	0.0079
B	0.5396	0.5124	0.5242	0.0136
C	0.681	0.6642	0.6789	0.0092
D	1.675	1.6432	1.6672	0.0166
E	1.1223	1.1124	1.1185	0.0050

**Table 5:** Comparison of observed photovoltaic characteristics with previously published findings.

Sl. No	Samples and Illumination Condition	V_oc_ (V)	J_sc_ (mA/cm^2^)	Fill Factor	Efficiency (%)	Ref
1	TiO_2_ coated Cds QDs (AM 1.5G irradiated with input solar power of 100 mW/cm^2^)	0.45	3.24	0.36	0.52	[32]
2	CdS QDs capped by PVA (AM 1.5G irradiated with input solar power of 100 mW/cm^2^)	0.53	4.40	0.30	0.69	[33]
3	TiO_2_/CNTs with CdS QDs (AM 1.5G irradiated with input solar power of 100 mW/cm^2^)	0.60	3.17	0.61	1.15	[34]
4	CdS QDs on Cu doped ZnO nanorods (AM 1.5G irradiated with input solar power of 100 mW/cm^2^)	0.74	2.26	0.68	1.59	[35]
5	Mn/Co cod-doped CdS QDs (AM 1.5G irradiated with input solar power of 100 mW/cm^2^)	0.37	6.73	0.67	1.67	Present Work

Further, the dopants of Mn and Co have significant impact on the energy transfer in the CdS quantum dots from host into Mn and Co with enhanced chare separation through non-radiative processes. The modified cascaded energy level structure within the bandgap of CdS led to sustained charge transport without the chemical interaction with the surroundings sustained stability. The dopants effectively led to the pathway for the photoelectrons generated from CdS quantum dots in to the determined acceptors. Further, for the device with the sample D shows better efficiency of 1.6750%, Mn^+2^(1%) and Co^+2^(2%) co-doped on CdS which means the co-dopant of Co is doubled the times of Mn has enhanced the transparency of CdS by shifting the absorption spectrum to a larger extent. Synergistic effect of Co and Mn has increased the charge accumulation in TiO_2_ and quantum dot interface with the specific concentration of 1% and 2%.

It is observed that, Mn and Co co-dopant has significant role in enhancing the performance of solar cell, which is 2.26% in the case of Mn and Co on CdTe quantum dots [28]. Followed, PCE reached to 6.00% for TiO₂/AlSe/CdS:Co(4%)/ZnS photoanode, compared to those of 4.69% and 3.85% for TiO₂/AlSe/CdS:Mn(4%)/ZnS and undoped TiO₂/AlSe/CdS/ZnS structures [29]. For carbon quantum dot based CdS doped with Co has produced an efficiency of 2.27% and 2.21% without Cobalt [30]. Similarly, doping of 1% Co on CdS led to the enhancement of performance of solar cell equal to 0.48% with maximum bandgap of 2.43 eV [31].

## Conclusion

The synthesis of Mn^+2^ and Co^2+^ co-doped CdS quantum dots and the X-ray diffraction study revealed the identical crystal structure without any deterioration of the properties of CdS. The particle size determined from XRD is intact with the TEM results for the size of the quantum dots ranging from 7 to 10 nm. The bandgap of the CdS quantum dot is influenced by the stoichiometry ratio of the presence of dopants in CdS. PL spectroscopy shows the strong emission peaks at 484 nm and 579 nm confirming the defects in the sample due to the presence of dopants. The maximum power conversion efficiency of 1.6750% is obtained for the solar cell with Mn(1%)/Co(2%) co-doped CdS in the photoanode of the solar cell with fill factor, open circuit voltage and short circuit current density of 0.6705, 0.3703 V and 6.7364 mA/cm^2^. It is concluded the CdS quantum dots with Mn and Co co-dopant has significant impact on the performance of Quantum Dot Sensitized Solar Cell.

## Data Availability

The datasets during and/or analysed during the current study are available from the corresponding author upon reasonable request.
